# Watch your step! A frustrated total internal reflection approach to forensic footwear imaging

**DOI:** 10.1038/srep21290

**Published:** 2016-02-16

**Authors:** J. A. Needham, J. S. Sharp

**Affiliations:** 1 School of Physics and Astronomy, University of Nottingham, Nottingham, NG7 2RD, UK

## Abstract

Forensic image retrieval and processing are vital tools in the fight against crime e.g. during fingerprint capture. However, despite recent advances in machine vision technology and image processing techniques (and contrary to the claims of popular fiction) forensic image retrieval is still widely being performed using outdated practices involving inkpads and paper. Ongoing changes in government policy, increasing crime rates and the reduction of forensic service budgets increasingly require that evidence be gathered and processed more rapidly and efficiently. A consequence of this is that new, low-cost imaging technologies are required to simultaneously increase the quality and throughput of the processing of evidence. This is particularly true in the burgeoning field of forensic footwear analysis, where images of shoe prints are being used to link individuals to crime scenes. Here we describe one such approach based upon frustrated total internal reflection imaging that can be used to acquire images of regions where shoes contact rigid surfaces.

Imaging footwear marks and cross matching shoe types does not provide as clear an identification of an individual as say fingerprint or DNA evidence. There are only a finite number of shoe types in circulation and only a small subset of these are found at crime scenes (this is particularly true in the UK). So how then, can we hope to use images of footwear that have been retrieved from a crime scene to differentiate between similar shoe types and identify an individual? The answer lies in the individual nature of the wear patterns that are exhibited by shoes worn by different people. A person’s gait determines how they distribute their weight when they walk and this results in different extents of mechanical wear being present at specific points on the soles of their shoes. These wear patterns are specific to the person wearing the shoes and could in principle be used as a means of identification. An excellent example of this was observed in a study performed by Fruchtenicht *et al.*^1^. These authors showed that the wear patterns formed on identical sets of boots worn by US marines during a training exercise, exhibited highly individual wear patterns. Although such wear patterns would not necessarily be able to identify a person as the perpetrator of a crime as readily as say fingerprint or DNA would, they could be used to link them (or at least their shoes) to a particular location–information that could be vital to law enforcement operatives.

A national footwear database (NFD) containing images of common shoe types has been developed in the UK to try to aid the processing of images that have been retrieved from crime scenes. The function of this database is very similar to that of the national fingerprint databases that currently exist in a number of countries in that it allows forensic scientists and practitioners to compare images of shoe prints obtained from crime scenes with a bank of images of known shoe types. It is also possible to compare images of shoeprints taken in custody, to marks that are found at crime scenes.

A key component in the process of forensic image comparison and analysis involves some method of collecting images of shoe prints from suspects. Previous studies have used fairly simple approaches based on commercially available footwear impression kits, where an ink pad (or reservoir of fluid), is used in conjunction with special sensitised paper to develop an image. This approach is very similar to that used during the retrieval of fingerprints. The resulting shoe prints are then typically scanned to digital format using flat-bed scanners^1^,^2^,^3^. Although this approach produces images of the contact regions in shoe prints that are of the required quality, the entire process is time consuming and can be costly in terms of the consumables (e.g. ink pads and sensitised paper) that are required. A more desirable approach is to use images acquired directly using digital methods.

The direct acquisition of images of shoeprints using digital techniques has, until now, presented a number of challenges. The most significant of these being that conventional illumination techniques (obtained using white light illumination) do not provide specific and detailed information about the regions where the shoe will contact a surface. However, there are a number of techniques that have been applied to measure surface contact between bare feet and a surface. These include the use of capacitive sensors, piezoelectric crystals, arrays of load cells and the mechanical deformation of elastomeric materials [see ref. 4 for a review]. One particular technique that has been used extensively for gait analysis and determining the distribution of contact points between feet and surfaces involves the use of frustrated total internal reflection (FTIR) imaging. This technique involves the use of an optical waveguide that is constructed from a sheet of glass, or plastic and on which the subject stands. It has been used successfully to image the contact regions of fingerprints^5^,^6^, as well as insect^7^,^8^,^9^, animal^10^ and human feet^11^ contacting hard surfaces. Its success in these areas has resulted in the development of imaging devices such as ‘CatWalk’ for measuring the gait of small animals^10^ and the ‘Multitoe’ direct touch controller^12^. FTIR imaging has also been used successfully in a number of microscopy related applications^13^,^14^.

In the FTIR technique, light is coupled into a transparent sheet of material by illumination from around its edges. This illumination is setup in such a way that the light is totally internally reflected inside the sheet–a condition which is achieved by ensuring that the light is always incident on the glass(plastic)/air interface at an angle that is larger than the critical angle, *θ*_*c*_. This angle can easily be determined from the formula 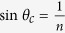, where *n* is the refractive index of the sheet material^15^. For glass, which has a refractive index of ~1.5 at visible wavelengths^16^, the critical angle is ~42°. Above the critical angle, the light is reflected back inside the sheet material because it has a higher refractive index than air. However, the boundary conditions at the sheet/air interface dictate that the electric field and its spatial derivatives are continuous across the interface. The result of this is that an evanescent field is present which decays exponentially away from the sheet material and into the air^15^. This evanescent field decays over length scales that are comparable to the wavelength of the illuminating light (400–700 nm for visible wavelengths) and is capable of interacting with objects that come within this range of the sheet surface. An object such as a foot, a finger, or even a shoe which comes close to the surface of the glass/plastic sheet will change the boundary conditions experienced by the light reflecting/refracting at the surface of the sheet material. In the case of a shoe sole, this change in boundary conditions can be thought of as changing the refractive index above the waveguide from that of air to that of the shoe material. As many shoes are typically made from polymeric materials, they will have a higher refractive index than air^17^. This change in refractive index results in a change in the critical angle required for light to be totally internally reflected at this interface which is now given by the equation 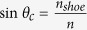, where *n*_*shoe*_ is the refractive index of the shoe material. The result of this change in refractive index, is that light in the regions where the shoe contacts the surface will no longer be totally internally reflected and will instead be transmitted into and scattered by the shoe sole. This scattering of light by the regions in contact (or close proximity) allows the contact regions to be detected and imaged.

## Methods

**Figure 1:**
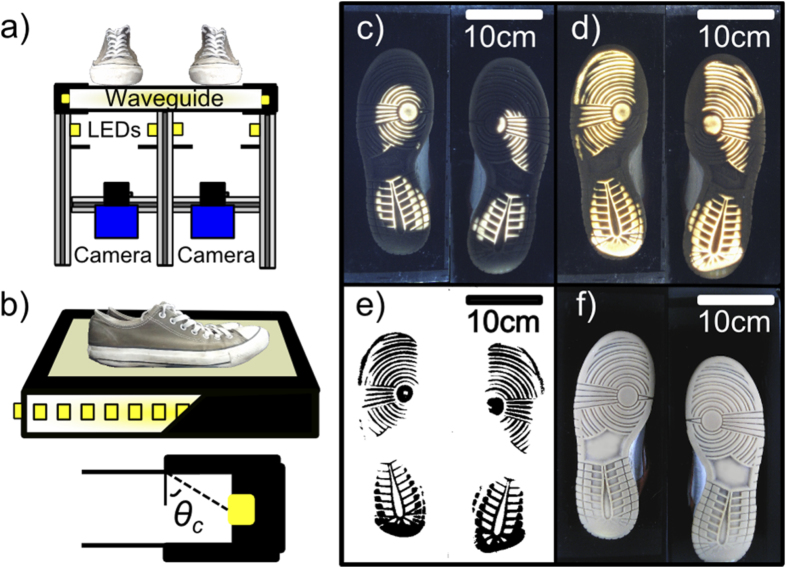
(**a**) Schematic diagram of the dual wave guide and conventional LED illumination apparatus for imaging shoe prints. (**b**) The waveguide is formed by wrapping ulltrabright LEDs around the perimeter of a thick glass (or perspex) sheet. The waveguide is masked so that only light incident on its surface at angles greater than *θ*_c_ is allowed to propagate. This light is confined inside the waveguide. (**c**) When a shoe contacts the waveguide surface, light is scattered strongly and can be imaged. (**d**) A gentle rocking motion of the shoes allows the wearer to mimic the walking action and a more complete image of the contact regions can be obtained. (**e**) The images in (**d**) are binarised to obtain a black and white representation of the contact regions of the shoes (black denotes the contact regions). (**f**) Conventional illumination can also be used to identify any defining features that are not in intimate contact with the waveguide surface.

The waveguide optical element used in FTIR illumination is made from a single piece of glass (or acrylic) that is 600 mm × 600 mm and 30 mm thick. Illumination of the waveguide element is achieved by wrapping ultrabright white LED strip lights around the roughened edge of the glass/acrylic sheet as shown in Fig. 1b. The LED strips are then held in place using black tape. This serves to secure the LED strip and to prevent ambient light from entering the waveguide at the edges. The edges of the two largest faces of the sheet are then masked using black tape (see Fig. 1b). This is done in such a way that light from the LEDs which subtends an angle of incidence less than the critical angle required for total internal reflection is prevented from escaping the waveguide sheet. As a result, only light with an angle of incidence greater than *θ*_*c*_ will be incident on the surface and will be confined within the waveguide as a result of total internal reflection. However, when a shoe comes into contact with the waveguide element, the condition for total internal reflection is changed and the contact regions are observed to strongly scatter the light. The shoes can then be imaged from below using the webcams (Fig. 1a,c,d).

To ensure that images of the entire sole print are captured, the webcams are each used to capture a short movie lasting only a few seconds, during which the person standing is asked to rock backwards and forwards, to mimic the action of walking (see movie S1 provided in supplementary information). The Python software then looks at each pixel in turn and extracts the largest intensity value measured by that pixel during the short movie sequence. In this way, images similar to that obtained in Fig. 1d can be obtained for the entire sole of the shoe (Fig. 1c). These images are then converted into black and white images (binarised, Fig. 1e) ready for comparison to the contact images stored on the national footwear database. This process is achieved by setting all pixels with an intensity greater than a chosen threshold value to 0 and values with intensities less than this value to 255 (8 bit image). In this way, the contact regions appear as black objects on a white background.

Conventional illumination is achieved using a second strip of LEDs mounted below the waveguide (see Fig. 1a). Images of shoes are collected using conventional illumination so that scuff marks, scratches and other marks that are not in contact with the surface can also be identified (Fig. 1f). The Python software is then used to alternate between conventional illumination and FTIR illumination via a series of simple relays that switch the power supplies of the two illumination sources on/off.

## Results and Discussion

**Figure 2:**
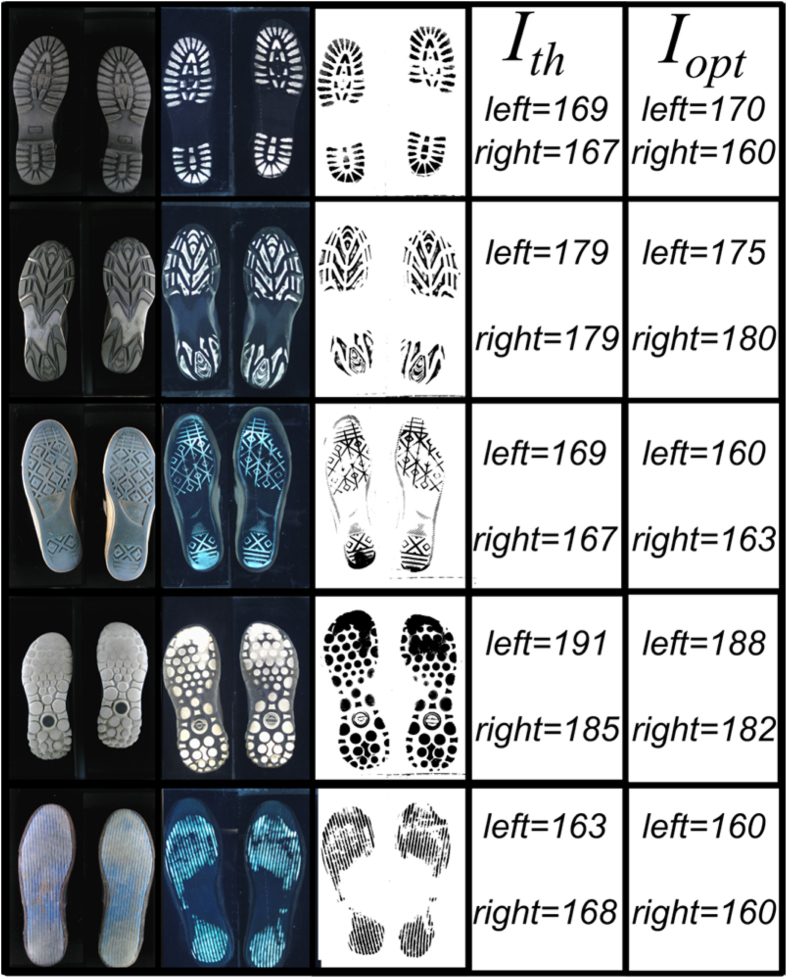
Examples of conventional (1^st^ column) and waveguide (2^nd^ column) images as well as the corresponding binarised image (3^rd^ column) obtained for different shoe types. The last two columns show the software determined threshold (*I*_*th*_) and the user optimised threshold (*I*_opt_) values used to produce the binarised images for each shoe (left and right).

The images shown in Fig. 1e,f are the most useful types of image to forensic scientists. The white light (conventional) image in Fig. 1f can be used to identify individual marks that are not present in the regions contacting a surface, but which may nevertheless be able to identify a person, from say an imprint impression on a soft surface. Meanwhile, the image obtained using the waveguide illumination technique (Fig. 1e) can be used to identify contact regions and corresponding wear patterns. Examples of images of different shoe soles that were obtained using our device are shown in Fig. 2, along with the corresponding contact images.

**Figure 3:**
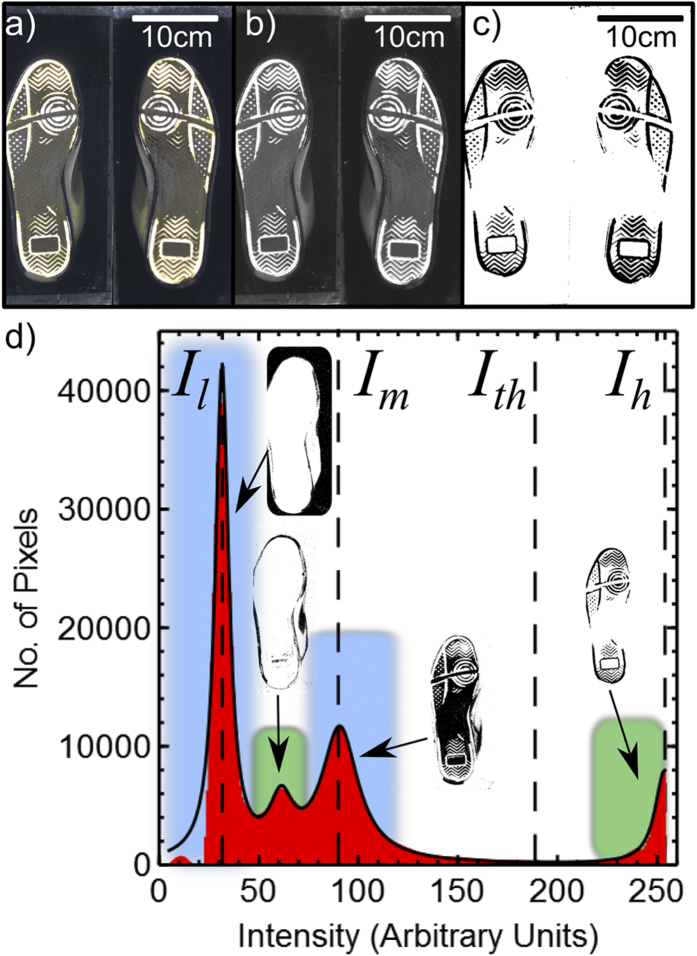
Thresholding the waveguide images in panel (a) involves converting the images to greyscale (panel (b)). The greyscale images are then binarised to form a black and white image of the contact regions (panel (**c**)). Panel (**d**) shows an example of an intensity histogram (red area) collected from the greyscale image of the left shoe shown in panel (**b**). Several peaks are present at different intensities corresponding to the background pixels (*I*_*l*_), the contact regions (*I*_*h*_) and regions of the shoe that are close to, but not in contact with, the waveguide surface (two peaks in the vicinity of *I*_*m*_). The solid black line in panel (**d**) is the result of a fit to the data using a sum of 4 Lorenztian peaks. The coloured rectangles and associated images show which peaks are associated with each of the features in the greyscale images. The threshold determined by the software is set to be at an intensity, *I*_*th*_, that is 60% of the way between *I*_*m*_ and *I*_*h*_ (see text).

The choice of threshold value used to binarise the images in Figs 1 and 2 is key in ensuring that a faithful representation of the contact regions is achieved. This process is semi-automated in the Python software used here, with the program selecting an initial guess for the threshold intensity value and then a user interface providing the operator with the ability to optimise this value. An example of how the automatic thresholding is achieved is shown in Fig. 3. The colour waveguide images acquired by the webcam (Fig. 3a) are initially converted to greyscale images (Fig. 3b) before being converted into binarised images (Fig. 3c). This binarisation process is performed by constructing an intensity histogram for the greyscale images by counting all the pixels at each intensity value (Fig. 3d). The data in this histogram is then fitted to a series of Lorentzian peaks. As Fig. 3 shows, the image histograms typically contain four peaks. The largest peak (centred at the lowest intensity value, *I*_*l*_) corresponds to the pixels in the background of the image as there are many more of these pixels than those obtained from the contact regions. The smaller peak at the highest pixel intensity value (*I*_*h*_ ~ *255*) corresponds to the regions of intimate contact between the shoe and the waveguide. Two additional peaks are also present close to an intermediate intensity value, *I*_*m*_ (Fig. 3d). These peaks correspond to regions of the image where the shoe sole is not in contact, but where it is close to the waveguide surface. The rectangles on the histogram and the corresponding images in Fig. 3d show which regions of the image (shown in black) are associated with each peak in the histogram.

The threshold value, *I*_*th*_ that is used to binarise the images is set by the software to be at a value 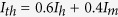. The operator/user is then given the opportunity to optimise this threshold value further for each shoe (left and right) separately, if required, by using slider controls on the user interface of the Python software. The intensity values quoted in Fig. 2 are for the threshold value chosen by the software (*I*_*th*_) and the final user determined threshold value used to binarise the images (*I*_*opt*_). These were found to be in good agreement for the majority of shoe types studied here. However, there are cases where, for example, the shoes are worn to the point where little of the tread pattern remains and the automatic threshold value does not quite work. Under these circumstances, a greater extent of user interaction is required to obtain the appropriate threshold value. It is worth noting at this point, that more sophisticated thresholding algorithms are available^2^,^3^,^18^. Although these could have been used here, the high quality of the images being collected and the favourable comparison of software and user defined threshold values indicates that our rather crude approach to thresholding works in most cases.

**Figure 4:**
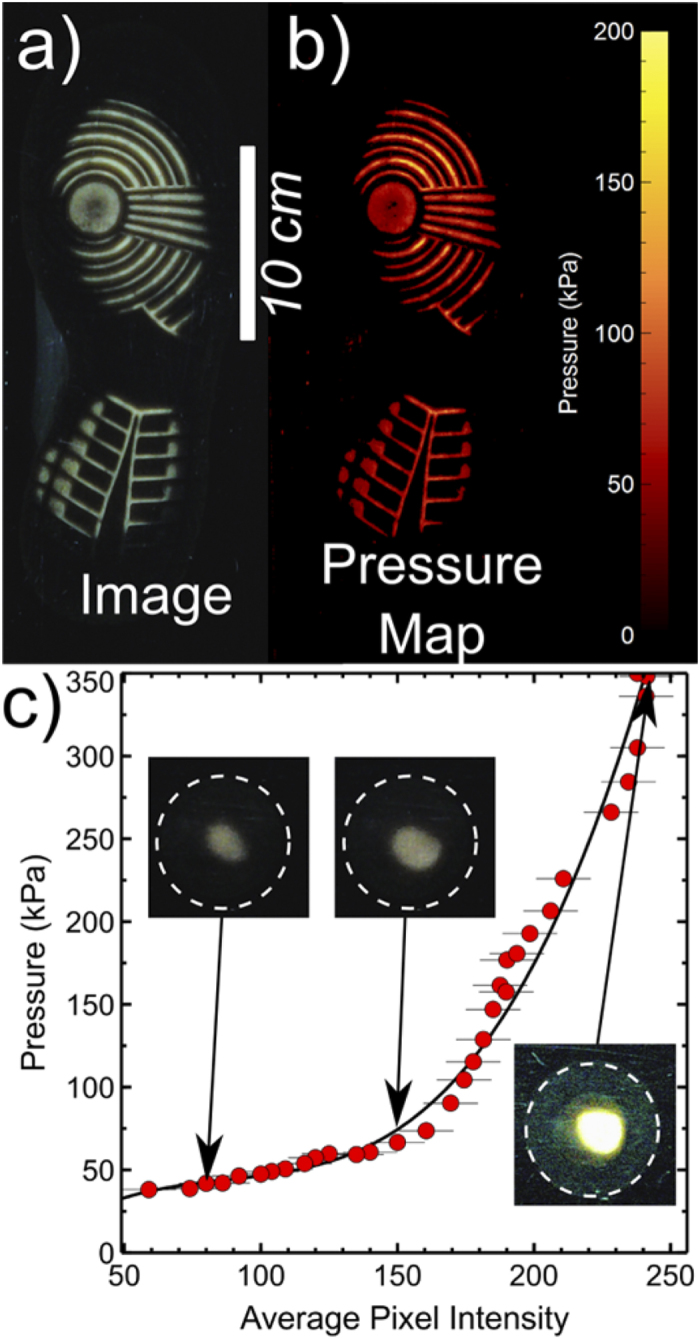
Pressure analysis of contact shoe print. Panel (**a**) shows a contact image obtained using waveguide illumination and panel (**b**) shows the corresponding pressure distribution that was obtained from this image. The conversion between panel (**a**) and panel (**b**) was performed using the calibration curve shown in panel (**c**), where measurements of applied pressure are related to the average pixel intensity in the contact regions. The solid line in this figure is the result of a 3^rd^ order polynomial fit to the data. The insets show waveguide contact images of the small rubber probe used in the calibration experiments (see text). The dashed circles mark the edge of the circular probe and show that only a small proportion of the probe is in contact.

More sophisticated analyses of the images obtained using waveguide illumination can be used to extract information about the distribution of pressure exerted by a person standing/walking on a hard surface^8^. Figure 4 shows an example of an image of a shoe print and its corresponding pressure map that have been extracted from one of the waveguide illumination experiments (panels a and b). Panel c in this figure shows the results of a calibration experiment, where a small round section cut from a similar shoe sole was placed on the waveguide surface and known loads/pressures applied to it. Waveguide images were obtained and used to create a calibration curve that relates the average local pixel intensity to the applied pressure. In obtaining the images in this figure, the exposure times of the webcams were set to be lower than those used to obtain the contact images in Figs 1, 2, 3. This was done to ensure that the intensity in the contact regions does not saturate when pressure is applied. Although saturation of the intensity values can be helpful in producing the simple contact images, variability in the pixel intensity with applied pressure is essential for determining how pressure is distributed underneath the sole of a shoe. The plot shown in Fig. 4c is representative of the pressure response across the surface of the waveguide in the regions being imaged. Repeat measurements found that the calibration plot shown in Fig. 4c was insensitive to position on the waveguide surface–a characteristic that is desirable for rapid conversion of the image intensity data to pressure values. A third order polynomial fit to the pressure-intensity plot shown in Fig. 4c was used to convert pixel intensities in the waveguide illumination images to pressure values and hence to convert Fig. 4a into Fig. 4b.

Care was taken during the calibration experiments to ensure that the true contact area was used in calculating the applied pressure. The insets shown in Fig. 4c reveal that only a small portion of the small probe was in contact with the waveguide surface. The area of this contact region was used in conjunction with the applied load to calculate an average pressure. These pressure data were plotted against the average value of the pixel intensities in this contact region and the errors in the intensity were determined from the full width at half height of the peak in the intensity histogram corresponding to the contact regions.

There are potentially interesting uses for this type of pressure analysis in forensic science. For example, this kind of approach could be used to determine how an individual distributes their weight through their shoe soles when they walk and/or run and hence determine roughly how fast they may have been moving when they deposited a shoe print. Combined measurements of contact area and pressure distributions could also be used to determine how hard an individual might have kicked a surface such as a door (during forced entry), or even possibly another person. Both of these would require slightly different imaging setups to the one described here. Clearly the use of a rocking motion to mimic walking/running used here has limited applicability. However, the imaging setup described here could be easily incorporated into a simple walkway/catwalk design and sunk into the floor for convenience of access and imaging. Similarly, a vertically mounted waveguide could be used to perform simulated kicking measurements. There may also be further applications for this imaging technique in clinical studies of gait analysis, or in measurements of how athletes interact with surfaces during high impact activities such as running, jumping, or changing direction. The simplicity of the technique lends itself to the study of many different types of activity and a range of environments.

## Additional Information

**How to cite this article**: Needham, J. A. and Sharp, J. S. Watch your step! A frustrated total internal reflection approach to forensic footwear imaging. *Sci. Rep.* **6**, 21290; doi: 10.1038/srep21290 (2016).

## Supplementary Material

Supplementary Information

Supplementary Movie S1
