# Supplementary material for: Watch your step! A frustrated total internal reflection approach to forensic footwear imaging
Source: Sci Rep. 2016 Feb 16;6:21290. doi: 10.1038/srep21290 (PMC4754702; doi:10.1038/srep21290)
Supplement: Supplementary Information [file srep21290-s1.pdf]

Watch your step! A frustrated total internal reflection approach to forensic footwear imaging

J.A. Needham and J.S. Sharp*

School of Physics and Astronomy, University of Nottingham, Nottingham, NG7 2RD, UK

Supplementary material

Movie S1 Caption: Movie showing a contact image of shoes on the waveguide surface as a person rocks backwards and forwards.
